# Determining Olefin Content of Gasoline by Adaptive Partial Least Squares Regression Combined with Near-Infrared Spectroscopy

**DOI:** 10.3390/molecules30244742

**Published:** 2025-12-11

**Authors:** Biao Du, Hongfu Yuan, Lu Hao, Yutong Wu, Chen He, Qinghong Wang, Chunmao Chen

**Affiliations:** 1 State Key Laboratory of Petroleum Pollution Control, College of Chemical Engineering and Environment, China University of Petroleum-Beijing, Beijing 102249, China; 2 CCIC YIXINGYUAN TECHNOLOGY (BEIJING) Co., Ltd., Beijing 101300, China

**Keywords:** adaptive partial least squares, near-infrared spectroscopy, gasoline, olefin

## Abstract

The accurate and rapid determination of olefin content in gasoline is crucial for fuel quality control. While near-infrared spectroscopy (NIR) offers a rapid analytical solution, multiple parameters in the conventional partial least squares regression (PLSR) modeling process rely on the modeler’s subjective judgment. Consequently, the quantitative accuracy of the model is often influenced by the modeler’s experience. To address this limitation, this study developed an integrated adaptive PLSR framework. The methodology incorporates four core adaptive components: automated selection of latent variables based on the rate of decrease in PRESS values, dynamic formation of calibration subsets using Spectral Angle Distance and sample number thresholds, optimization of informative wavelength regions via correlation coefficients, and systematic database cleaning through iterative residual analysis. Applied to 248 gasoline samples, this strategy dramatically enhanced model performance, increasing the coefficient of determination (*R*^2^) from 0.7391 to 0.9102 and reducing the root mean square error (*RMSE*) from 1.51% to 0.866% compared to the global PLSR model. This work demonstrates that the adaptive PLSR framework effectively mitigates spectral nonlinearity and improves predictive robustness, thereby providing a reliable and practical solution for the on-site, rapid monitoring of gasoline quality using handheld NIR spectrometers.

## 1. Introduction

As one of the most important automotive fuels worldwide, gasoline quality directly impacts engine performance, exhaust emissions, and ambient air quality [1,2,3]. Among various gasoline quality indicators, olefin content is a critical parameter requiring strict control [4,5]. Chemically reactive olefins tend to form deposits within engines, leading to injector clogging and valve sticking, which subsequently increases carbon buildup, reduces combustion efficiency, and elevates pollutant emissions [6,7,8]. Therefore, establishing rapid and accurate methods for olefin content detection is of paramount importance for fuel quality control and environmental protection.

Gas chromatography [9] and fluorescence indicator adsorption [10] are established as standard methods for determining olefin content in gasoline. While these methods provide accurate and reliable results, they are associated with lengthy analysis times and a dependence on laboratory settings, making them unsuitable for rapid screening and real-time monitoring of fuel quality at sites such as gas stations and oil depots. Additionally, direct analysis in real-time mass spectrometry has been applied to the analysis of crude oil and petroleum fractions [11]. However, due to limitations such as low selectivity towards unmodified or unlabeled compounds like olefins, bulky equipment, and high cost, it remains challenging to achieve rapid detection of olefin content in petrol. In contrast, near-infrared spectroscopy (NIR) has gained widespread use in the field of rapid fuel quality analysis due to its notable advantages, including high analysis speed, minimal sample preparation, and non-destructive measurement [12,13,14]. By integrating chemometric techniques, quantitative calibration models can be developed to correlate spectral data with target properties, such as olefin content, enabling rapid prediction of sample characteristics [15].

Partial least squares regression (PLSR) has become the predominant modeling method in near-infrared spectroscopy due to its effectiveness in handling spectral multicollinearity while utilizing information from both spectral and property matrices [16,17,18]. However, as a linear method, PLSR performance relies heavily on the alignment between calibration samples and unknown samples [19]. In practical applications, especially with gasoline samples from diverse refineries, variations in crude sources and processing technologies create broad compositional distributions. This leads to significant nonlinearity between spectral responses and olefin content, substantially compromising the accuracy and robustness of conventional global PLSR models and limiting their potential for on-site rapid detection [20].

The adaptive modeling strategies present a promising approach for addressing the limitations of linear PLSR models when analyzing complex gasoline samples [21]. The method dynamically constructs representative sample subsets from large spectral databases based on spectral similarity, enabling accurate capture of local structure-property relationships while mitigating distortions caused by global nonlinearities [22]. Although previous research has explored spectral variable selection and model transfer techniques [23,24,25,26], a fully automated and systematic adaptive PLSR framework for quantifying gasoline olefin content via near-infrared spectroscopy requires further development [27]. Key challenges include the automated optimization of calibration set composition, appropriate determination of latent variables, selection of informative spectral regions, and implementation of effective quality control measures for modeling data, all of which demand comprehensive investigation and integrated solutions.

Therefore, this study aims to systematically develop and validate an adaptive PLSR methodology for rapid determination of olefin content in gasoline. Based on 248 gasoline samples whose near-infrared spectra were collected and olefin contents were determined by gas chromatography, this research focuses on four critical aspects: adaptive determination of latent variables, dynamic selection of modeling samples, optimization of characteristic wavelengths, and quality control procedures for calibration datasets. By integrating these adaptive modules, we expect to significantly enhance the prediction accuracy and robustness of handheld near-infrared spectrometers when analyzing gasoline samples from diverse sources and with broad property ranges. The proposed framework will provide reliable technical support for efficient and effective olefin content monitoring in complex field environments such as gas stations and oil depots.

## 2. Experimental Section

### 2.1. Database Collection

A total of 248 commercial gasoline samples conforming to China’s National VI standard were collected from multiple sources, including Sinopec gas stations, Shunyi Niushan Oil Depot, and regional market surveillance samples, covering both RON 92 and RON 95 grades. Spectral acquisition was performed using a self-developed handheld near-infrared analyzer configured with 32 scans per measurement. Each sample was analyzed in triplicate, and the average spectrum was adopted as the final spectral representation. The olefin content of the gasoline samples was determined as reference values for spectral calibration, following the standard method GB/T 30519-2014, “Determination of Hydrocarbon Family Composition and Benzene in Light Petroleum Fractions and Products (Multidimensional Gas Chromatography Method)”.

### 2.2. Data Processing

All data processing was performed in MATLAB R2024R. Spectral baseline drift was corrected by applying the Savitzky–Golay first-derivative transformation. The linear correlation coefficient between the preprocessed NIR spectra and the reference olefin content was subsequently computed. Custom scripts were developed to implement four adaptive procedures: selection of latent variables, calibration set formation, wavelength region optimization, and sample data cleaning. Finally, the predictive performance of global PLSR modeling was systematically compared with that of the proposed adaptive PLSR approach. The model performance evaluation parameters used are as follows.

Coefficient of determination (Equation (1)):

(1) $$R^{2}=1-\frac{{\sum_{i=1}^{n}\left(y_{i}^{p}-y_{i}^{ref}\right)}^{2}}{{\sum_{i=1}^{n}\left(y_{i}^{ref}-{\bar{y}}^{ref}\right)}^{2}}$$

$R^{2}$ represents the coefficient of determination for prediction, n stands for the number of samples, $y_{i}^{p\;}\;$ for the predicted value of the *i*-th sample, $y_{i}^{ref}$ for the reference value of the *i*-th sample, and ${\bar{y}}^{ref}$ for the mean of the reference values.

Root Mean Square Error (*RMSE*, Equation (2)):

(2) $$RMSE=\sqrt{\frac{1}{n}\sum_{i=1}^{n}{\left(y_{i}-\hat{y_{i}}\right)}^{2}}$$

*n* represents the number of samples, $y_{i}^{p}$ stands for the predicted value of the *i*-th sample, $y_{i}^{ref}$ for the reference value of the *i*-th sample.

## 3. Results and Discussion

### 3.1. Spectral Characteristic Analysis

**Figure 1:**
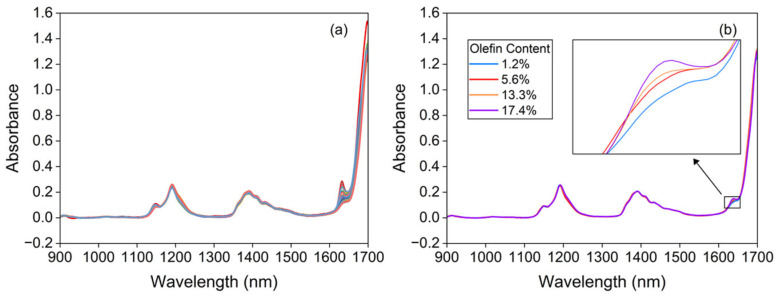
Near-infrared spectra of the 248 petrol samples collected (**a**) and petrol with varying olefin content (**b**).

Near-infrared spectroscopy belongs to the overtones and combination bands of the fundamental vibrational frequencies of molecular vibration spectra. Hydrogen-containing functional groups (C-H, O-H, N-H, S-H) all exhibit absorption peaks within this region. As petrol comprises a multitude of hydrocarbon compounds, the methyl, alkene, methylene, and aromatic hydrocarbon moieties within these hydrocarbons display abundant characteristic absorption spectra in the near-infrared region. In the near-infrared spectrum of olefin molecules, the most prominent characteristic peak originates from the terminal methylene C-H bond associated with vinyl and vinylidene groups [28]. The spectral range around 1620–1640 nm corresponds to the first CH stretching over-tone [29], while 1100–1300 nm corresponds to the second overtone of the CH stretching vibrations [28]. Figure 1a displays the collected spectra of 248 petrol samples with varying olefin contents. Figure 1b presents the near-infrared spectra of four representative samples (1.2%, 5.6%, 13.3%, and 178.4% olefin content) within the 1620–1640 nm range, as the olefin content in the samples increases, the absorption curves of the spectral peaks become steeper, indicating greater absorbance. This demonstrates a positive correlation between the absorbance of the characteristic olefin peaks in petrol and their respective olefin content.

### 3.2. Assessment of Nonlinearity

To assess the degree of linear correlation between the near-infrared spectra of gasoline samples and their olefin content, correlation coefficients were computed between the absorbance at each wavelength and the olefin content across all 248 gasoline samples (Equation (3)).

(3) $$R_{j}=\frac{\sum_{i=1}^{n}(x_{i}-\bar{x})(y_{i}-\bar{y})}{\sqrt{\sum_{i=1}^{n}{\left(x_{i}-\bar{x}\right)}^{2}\sum_{i=1}^{n}{\left(y_{i}-\bar{y}\right)}^{2}}}$$

Here, R denotes the correlation coefficients at the jth wavelength point. $\bar{x}=\frac{\sum_{i=1}^{n}x_{i}}{n}$, $\bar{y}=\frac{\sum_{i=1}^{n}y_{i}}{n}$, $x_{i}$ denotes the absorbance of the *i*-th sample at the *j*-th wavelength, $y_{i}$ denotes the olefin concentration corresponding to the *i*-th sample, and n denotes the number of samples.

**Figure 2:**
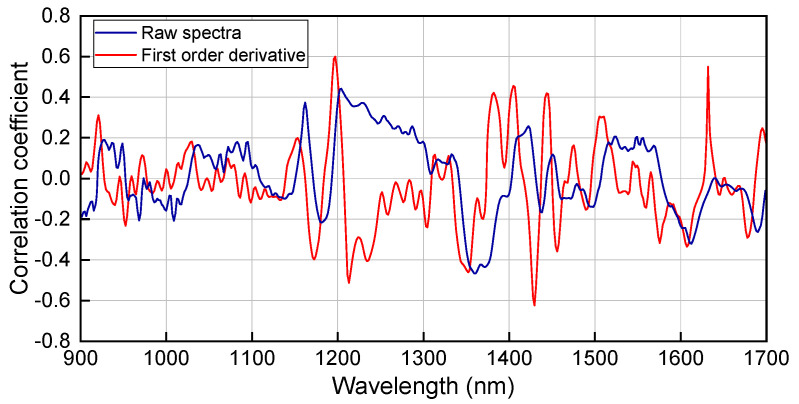
Correlation coefficient curves between the olefin content and the spectra (original and first-derivative).

A stronger linear relationship is indicated by an absolute correlation coefficient closer to 1. Figure 2 presents the correlation coefficient curves derived from the raw spectra and their first derivatives in relation to olefin content. The maximum absolute correlation coefficient obtained from the raw spectra remains below 0.5. In contrast, following first-derivative preprocessing, the correlation coefficients at 1204 nm and 1429 nm exceed 0.6 in absolute value. The spectral range around 1204 nm corresponds to the second overtone of the CH stretching vibrations, while 1429 nm corresponds to the first overtone of the OH stretching vibrations. These findings demonstrate that the first-derivative processing enhances the linear correlation between spectral features and olefin content. Nevertheless, the fact that the maximum absolute correlation coefficient remains substantially lower than 1 underscores a marked nonlinear relationship between the spectral data and olefin content in the studied dataset.

### 3.3. Selection Strategy for Latent Variable Number in Adaptive Modeling

In the application of adaptive partial least squares (PLS) methods, it is necessary to construct an individual calibration model for each sample to predict its properties, wherein the number of latent variables (LVs) is also determined adaptively per sample. The selection of the number of LVs is critical, as too few may result in underfitting, while too many can lead to overfitting, thereby significantly influencing model performance. Conventionally, the optimal number of LVs is chosen by analyzing the curve of the prediction residual sum of squares (PRESS) versus the number of LVs in the calibration set, typically selecting the number corresponding to the minimum PRESS value. However, PRESS curves can exhibit diverse patterns, such as monotonic decrease, decrease followed by an increase, initial increase, mid-range fluctuations, or abrupt variations, which makes selection based solely on the minimum PRESS value susceptible to overfitting.

To overcome this limitation, an adaptive selection strategy is proposed based on the rate of decrease in the PRESS values. This method identifies the optimal number of latent variables by evaluating the decline rates across four predefined segments of the PRESS curve: (1) three consecutive points from the start to the end of the curve, (2) three consecutive points containing only the lowest PRESS value, (3) four intermediate points including the minimum, and (4) two adjacent points encompassing the lowest point. A decline rate threshold is applied to guide the selection. Moreover, the relationship between the decline rate threshold and both *R*^2^ and root mean square error (*RMSE*) is analyzed by plotting corresponding curves. The optimal threshold, yielding the maximum coefficients of determination (*R*^2^) or the minimum *RMSE*, is then used to determine the final number of principal factors for modeling.

**Figure 3:**
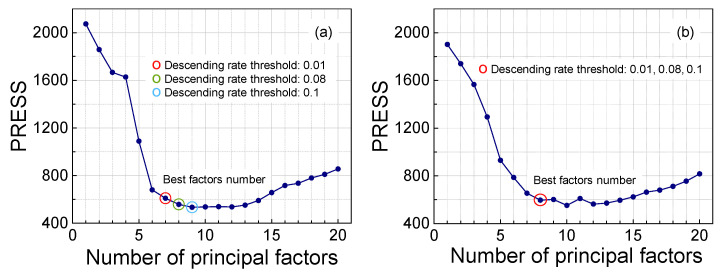
For the PRESS curve calculated using the (**a**) raw and (**b**) first derivative spectra. The optimally selected number of principal factors by adaptation when the descending rate thresholds are 0.01, 0.08, and 0.1.

To evaluate the efficacy of the proposed strategy, validation was carried out using raw spectra and olefin content data from 248 gasoline samples. For models based on raw spectra, the minimum PRESS value was attained at the 9th principal component, with the curve remaining stable in its vicinity (Figure 3a). When different descending rate thresholds (0.01, 0.08, and 0.1) were applied, the adaptive strategy selected 9, 8, and 7 latent variables, respectively, revealing a consistent decrease in the number of principal components as the threshold increased. In the PLS model constructed from first-derivative spectra, the global PRESS minimum occurred at the 10th principal component (Figure 3b), accompanied by notable fluctuations nearby. Under the different descending rate thresholds (0.01, 0.08, and 0.1), the method consistently identified the 8th latent variable as optimal. Although the PRESS value at this point was higher than the absolute minimum, it was lower than that of neighboring points, confirming the robustness of the strategy in the presence of complex PRESS trends.

**Figure 4:**
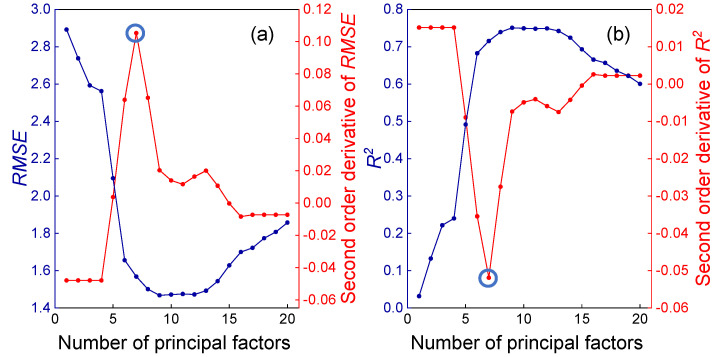
*RMSE* (**a**) and *R*^2^ (**b**), along with their second derivatives, versus the number of principal factors.

Furthermore, based on the reference values and cross-validated predicted values from all samples, *R*^2^ and *RMSE* were analyzed against different numbers of principal factors. For the PRESS curve obtained from the raw spectra, the extreme points in the second derivatives of both *RMSE* and *R*^2^ occurred at the 7th latent variable (Figure 4a,b), indicating an inflection point where the trend transitions from rapid change to gradual stabilization. When a descent rate threshold of 0.1 was applied, the adaptive strategy also selected the 7th principal factor (Figure 3a). A comparative analysis reveals that while the 7th factor corresponds to the inflection point, which may indicate underfitting, the 9th factor aligns with the minimum PRESS value, carrying a risk of overfitting. A balanced choice at the 8th principal factor (Figure 3) thus offers a compromise between underfitting and overfitting for the model based on raw spectral data.

### 3.4. Adaptive PLSR Calibration Set Selection Strategy

When developing PLSR models using NIR spectral data and gasoline olefin content, two fundamental principles merit consideration: first, samples with similar spectral profiles tend to exhibit comparable chemical compositions; second, a sufficient number of representative calibration samples is essential to ensure statistical validity. Guided by these principles, this study proposes an adaptive calibration set selection strategy for PLSR modeling.

The Spectral Angle Distance (SAD) function is employed to evaluate the similarity between spectral profiles. For each individual sample, SAD values are sequentially computed against all other samples in the dataset. Those samples with SAD values below a predefined threshold are identified as adjacent samples. The selected similar samples form the calibration set, and the model calibrated using this set demonstrates enhanced suitability for predicting the target sample. Within the adaptive strategy, one sample is randomly selected from the dataset. By applying both the SAD threshold and a calibration sample quantity threshold, a group of adjacent samples is filtered from the database to constitute the calibration set. Subsequently, following the adaptive latent variable number selection strategy described in Section 3.2, a PLSR model for olefin content is established. This model is then used to predict the olefin content of the target sample, after which the sample is returned to the database. This procedure is repeated for every sample in the database, ensuring each is selected exactly once. Based on the predicted and reference values from all samples, the *R*^2^ and *RMSE* are calculated. These metrics are used to systematically evaluate model performance under varying SAD thresholds and calibration set size thresholds.

(1) SAD threshold

**Figure 5:**
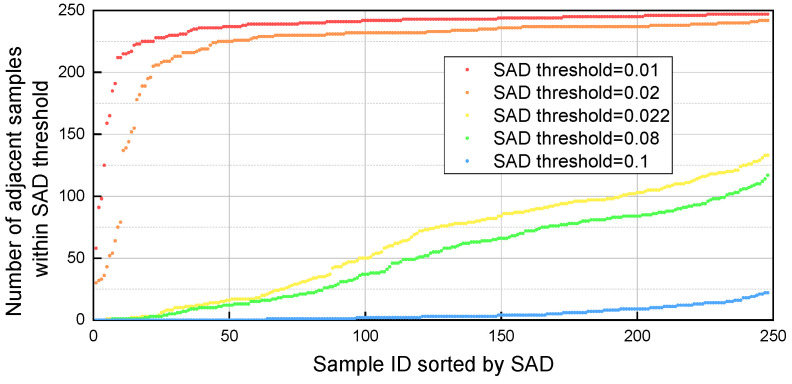
Number of adjacent samples for each sample within five different SAD thresholds.

Figure 5 illustrates the distribution of adjacent sample counts for each gasoline sample in the dataset under five distinct SAD threshold ranges. As expected, the number of adjacent samples varies significantly across different samples and demonstrates a strong dependence on the SAD threshold: lower thresholds result in fewer adjacent samples. Notably, when the SAD threshold exceeds 0.08, the majority of samples in the database possess more than 200 adjacent samples, indicating a substantial expansion of the potential calibration set size beyond this threshold value.

**Figure 6:**
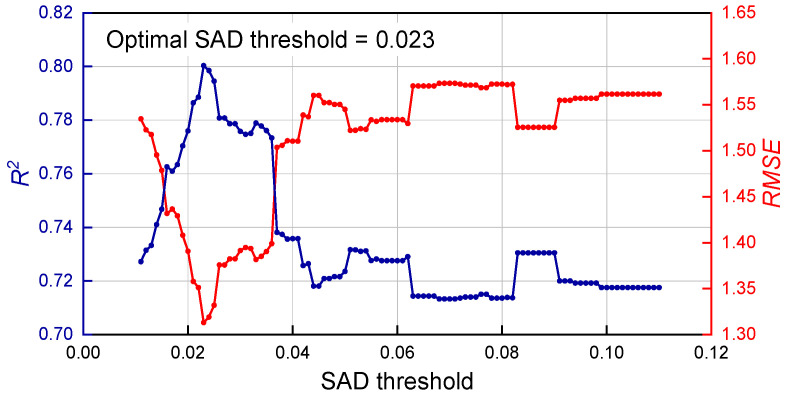
Curves of *R*^2^ (blue) and *RMSE* (red) varying with the SAD threshold.

After setting the SAD threshold, for any individual sample in the database comprising 248 gasoline samples, the remaining samples are categorized into two classes: the sample-sufficient class and the sample-insufficient class. Based on extensive exploratory experiments, the thresholds for the number of modeling samples—namely, threshold of calibration sample number for sufficient data (TCSNSD) and threshold of calibration sample number for insufficient data (TCSNID)—were initially set to 107 and 116, respectively. This setting was determined through preliminary experiments in which, due to the interdependent relationship among SAD, TCSNSD, and TCSNID, two parameters were fixed at a time to systematically optimize the third. It was found that across the SAD threshold range of 0.01–0.11, the optimal values for TCSNSD and TCSNID consistently remained 107 and 116. Therefore, these two parameters were fixed at 107 and 116. Subsequently, adaptive selection of the modeling calibration set was performed within the SAD threshold range of 0.01–0.11 (in increments of 0.001), and the *R*^2^ and *RMSE* values under different SAD thresholds were calculated. Figure 6 shows the curves of (a) *RMSE* and (b) *R*^2^ as functions of the SAD threshold, indicating significant fluctuations in both metrics. The *R*^2^ value increased sharply with the SAD threshold, reaching a peak of 0.8 at 0.023, after which it generally declined. In contrast, the *RMSE* exhibited an opposite trend. Overall, the SAD threshold of 0.023, corresponding to the maximum *R*^2^ value, was identified as the optimal threshold.

(2) Threshold of Calibration Sample Number

**Figure 7:**
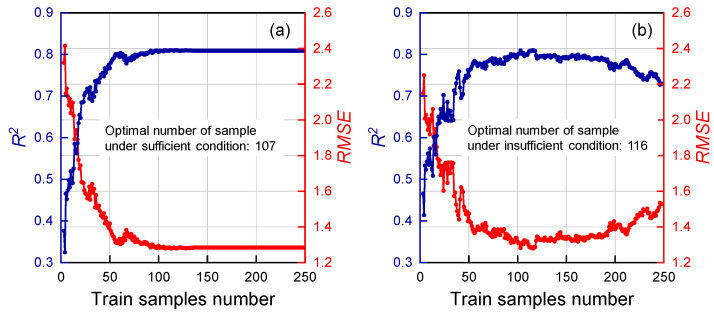
Curves of *R*^2^ (blue) and *RMSE* (red) versus TCSNSD (**a**) and TCSNID (**b**).

Following a procedure analogous to the adaptive SAD threshold selection, the SAD threshold was fixed at 0.023, and the calibration sample number threshold under insufficient data conditions (TCSNID) was set to 116. The *R*^2^ and *RMSE* values were then computed across a TCSNSD range from 1 to 247 (the total number of samples being 248), with an increment of 1. As shown in Figure 7a, the variations of *R*^2^ and *RMSE* as functions of TCSNSD are presented, respectively. Based on these results, the optimal TCSNSD was identified as 107.

Similarly, with the SAD threshold maintained at 0.023 and TCSNSD fixed at 107, the *R*^2^ and *RMSE* values were evaluated for models constructed under different TCSNID values. Figure 7b depict the corresponding trends of *RMSE* and *R*^2^ with respect to TCSNID. The analysis determined the optimal TCSNID to be 116. Beyond this value, *R*^2^ exhibited a slight decline but overall remained stable, whereas *RMSE* showed a minor increase before also stabilizing.

(3) Evaluation of Global and Adaptive Sample Modeling Strategies

**Figure 8:**
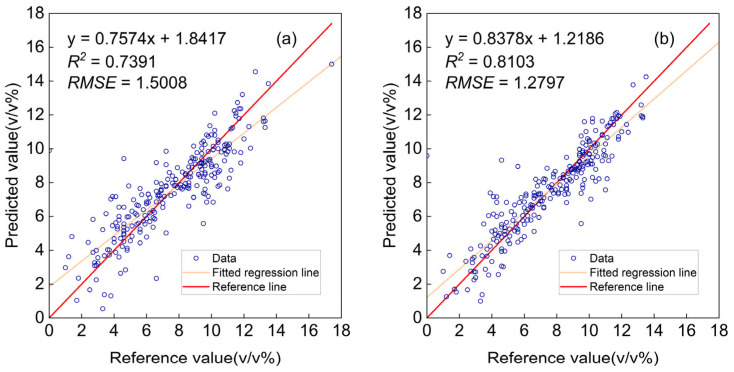
Predicted versus reference values: (**a**) modeling using all samples; (**b**) modeling with adaptive sample selection.

Through the aforementioned optimization process, the optimal parameters—SAD threshold, TCSNSD, and TCSNID—were determined as 0.023, 107, and 116, respectively. Figure 8a,b, respectively, compare the predicted values against reference values (measured by gas chromatography) for models constructed using the entire sample set versus those built with adaptive sampling. The olefin content of the gasoline samples was determined as reference values for spectral calibration, following the standard method GB/T 30519-2014, “Determination of Hydrocarbon Family Composition and Benzene in Light Petroleum Fractions and Products (Multidimensional Gas Chromatography Method)”. The results demonstrate that adaptive sample selection markedly enhanced the predictive performance of the model, evidenced by an increase in *R*^2^ from 0.7391 to 0.8103 and a decrease in *RMSE* from 1.51% to 1.28%. The above results indicate that through intelligent selection of a calibration set which balances spectral similarity with statistical representativeness, both model accuracy and generalizability are substantially enhanced. This approach offers a clear advantage over global modeling using all available samples, as it effectively mitigates the influence of spectrally dissimilar or outlier samples on the calibration model.

### 3.5. Adaptive Selection of Modeling Wavelengths

Conventional PLSR modeling using full-spectrum data often incorporates uninformative or noisy variables, which can degrade model interpretability and predictive performance. To address this limitation, an adaptive wavelength selection strategy was implemented to identify and retain only the most informative spectral regions.

To investigate the influence of adaptive wavelength selection on the performance of the PLSR model, the raw spectra of gasoline samples were employed for modeling. Spectral wavelength intervals with correlation coefficients above a specified threshold were retained for analysis. The modeling was conducted using the previously optimized parameters: a SAD threshold of 0.023, TCSNSD of 107, and TCSNID of 116. The impact of the selected wavelength intervals on model performance was assessed using the coefficient of determination (*R*^2^), following the procedure outlined below.

**Figure 9:**
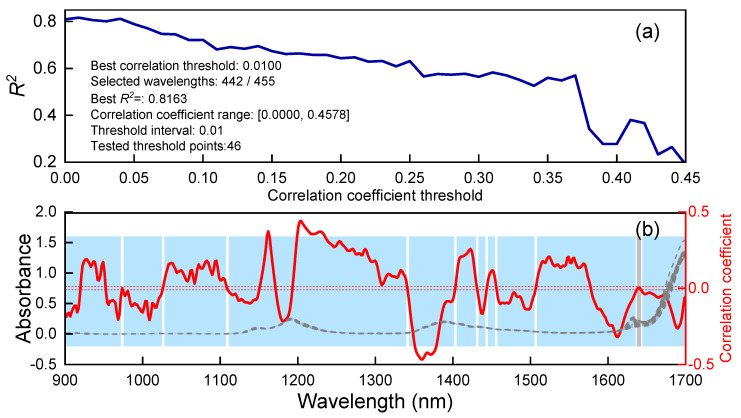
Curve of *R*^2^ versus correlation coefficient threshold (**a**). Adaptively selected wavelength region (blue) (**b**).

The correlation coefficient threshold between the spectra and olefin content was systematically increased from 0 to 0.45 in increments of 0.01. For each threshold, relevant wavelengths were selected and used to develop corresponding PLSR models. As illustrated in Figure 9a, the relationship between *R*^2^ and the correlation coefficient threshold shows a peak *R*^2^ value of 0.8163, slightly higher than that achieved in Figure 8b (*R*^2^ = 0.8103). The optimal correlation coefficient threshold was determined to be 0.01, at which 442 out of the total 455 wavelengths were retained. These selected wavelengths, highlighted in blue in Figure 9b, define the final modeling spectral region.

### 3.6. Data Quality Control Strategy for Database

As is well known, the quality of the modeling sample data critically influences model performance. Data quality control of sample sets is also an important part of the adaptive PLSR method. The data quality of a sample set is governed by three principal factors: the representativeness of sample distribution, the accuracy of spectral data, and the reliability of reference values. Together, these factors may introduce samples with non-negligible errors into the dataset, thereby undermining model predictive ability.

(1) Sample set cleaning strategy

To enhance data quality in a systematic manner, this study introduces a sample set cleaning strategy. First, spectral dimensionality reduction and visualization techniques are applied to identify potential outlier samples, which are re-measured when feasible for verification. Next, the adaptive PLSR modeling approach is utilized to compute prediction residuals for each sample and the overall coefficient of determination (*R*^2^). An iterative cleaning procedure is then carried out, whereby samples with the largest prediction deviations are sequentially removed, and model performance is re-evaluated after each removal. This process continues until the maximum absolute prediction deviation falls below the reproducibility limit of the reference method. By integrating spectral characteristics with modeling outcomes, this approach provides a systematic framework for data cleaning and facilitates the development of robust PLSR models.

(2) Distribution Characteristics of Gasoline Sample Sets

**Figure 10:**
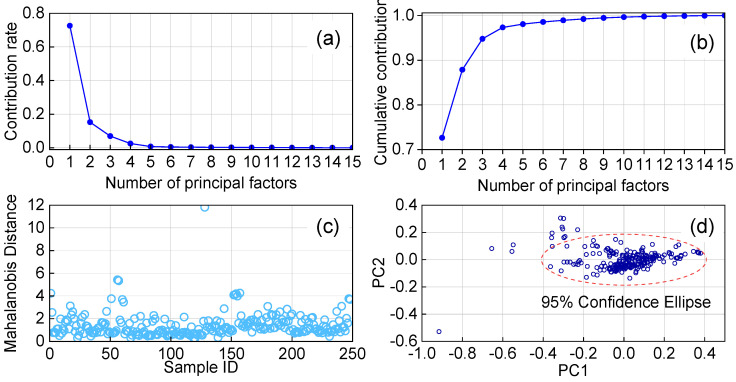
Contribution rate of principal components (**a**); Cumulative contribution rate of principal components (**b**); Distribution of Mahalanobis distances of samples (**c**); Distribution of samples in the principal component space (**d**).

Figure 10 presents the principal component analysis (PCA) results of near-infrared spectral data obtained from 248 gasoline samples. As shown in Figure 10b, the cumulative contribution rate of the first seven principal components reaches 98.93%, indicating that these components effectively capture the majority of spectral variance. Figure 10c reveals that 26% of the samples exhibit a Mahalanobis distance within 2.65, while just one sample is identified as a pronounced outlier located far from the main cluster. The overall spatial distribution of samples, depicted in Figure 10d, shows varying density across the feature space.

(3) Effectiveness Evaluation of the Sample Set Cleaning Strategy

**Figure 11:**
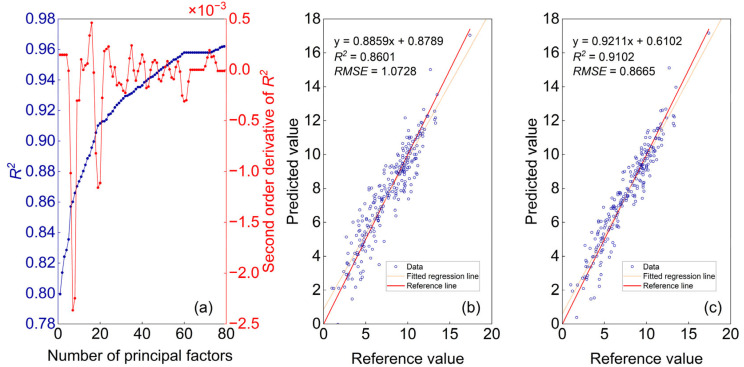
Curves of *R*^2^ and its second derivative versus the number of sample removals (**a**); Predicted versus reference values: modeling results after removing the first 7 samples (**b**) and modeling results after removing the first 19 samples (**c**).

A PLSR model was established using an initial training set of 248 gasoline samples. Residuals for each sample were calculated based on cross-validated predictions and reference values. The sample with the largest absolute residual was iteratively removed to form an updated training set. This elimination procedure was repeated until the maximum absolute residual fell below the reproducibility limit (1.2%) specified in GB/T 30519-2014. Figure 11a shows the variation in *R*^2^ and its second derivative with respect to the number of samples removed. The second derivative curve exhibits its first minimum at 7 removals and a second minimum at 19 removals. Between the start and 7 removals, and again between 7 and 19 removals, the *R*^2^ curve rises sharply, suggesting that the removed samples had suspect data quality. Beyond 19 removals, the *R*^2^ curve begins to plateau. Figure 11b,c present the modeling results after removing the first 7 and the first 19 samples, respectively. The corresponding *R*^2^ values improved from the initial 0.8 to 0.86 and 0.91, while the *RMSE* decreased from 1.28% to 1.07% and 0.866%, respectively, demonstrating a clear enhancement in model performance.

## 4. Conclusions

This study systematically addressed the limitations of conventional global PLSR in NIR quantitative analysis by developing an integrated adaptive PLSR framework. The proposed methodology incorporates four core adaptive components: latent variable number selection, calibration subset formation, informative wavelength region optimization, and systematic database cleaning. Using the determination of olefin content in gasoline as a case study, the framework demonstrated significant performance improvements over the standard full-sample PLSR modeling approach. The *R*^2^ increased substantially from 0.7391 to 0.9102, while the *RMSE* decreased correspondingly from 1.51% to 0.866%, confirming a marked enhancement in predictive accuracy. By implementing a systematic, data-driven strategy for optimizing critical modeling parameters, this work not only advances the mechanistic application of PLSR in NIRS but also establishes a practical and effective framework for improving quantitative analysis workflows across various domains of near-infrared spectroscopy. In the future, we will further enhance the reliability of the model by increasing the number of representative modeling samples, improving the accuracy of the reference data, enhancing the quality of spectral detection, selecting wavelength regions more closely related to olefin content, and applying more suitable pre-processing methods to eliminate the influence of irrelevant signals on the model.

## Data Availability

The raw data supporting the conclusions of this article will be made available by the authors on request.
